# Sulforaphane alleviates hypoxic vestibular vertigo (HVV) by increasing NO production via upregulating the expression of NRF2

**DOI:** 10.1080/21655979.2022.2030592

**Published:** 2022-04-20

**Authors:** Liyuan Zhou, Changchen Hu, Yujun Li, Binquan Wang

**Affiliations:** a Department of Otolaryngology, Head Neck Surgery, The First Hospital, Shanxi Medical University, Taiyuan, Shanxi, China; b Shanxi Key Laboratory of Otorhinolaryngology Head and Neck Cancer, Taiyuan, Shanxi, China; c Department of Neurosurgery, Shanxi Provincial People's Hospital, Shanxi Medical University, Taiyuan, China; d Department of Neurosurgery, Shuozhou People's Hospital, Shuozhou, China

**Keywords:** HVV, sulforaphane, NO, cGMP, NRF2, oxidative stress

## Abstract

Sulforaphane (SFP) treatment represses oxidative stress by activating NRF2. Meanwhile, SFP may also increase the production of nitric oxide (NO) and activate the signaling pathway of cyclic guanosine monophosphate (cGMP), which is involved in the pathogenesis of hypoxic vestibular vertigo (HVV). However, it remains unknown as whether SFP plays a therapeutic role in the treatment of HVV. A rat model of HVV was established to measure the levels of escape latency, malondialdehyde (MDA), glutathione (GSH) and superoxide dismutase (SOD) in the aorta tissues. Quantitative real-time PCR was performed to evaluate the expression of NRF2 mRNA, and Western blot and immunohistochemistry were carried out to analyze the expression of NRF2 protein. ELISA was used to examine the production of NO and cGMP. SFP treatment helped to maintain the escape latency and MDA, GSH, SOD concentrations in the brain of HVV rats, and recovered the expression of NRF2 inhibited in the brain of HVV rats. SFP treatment also elevated NO and cGMP production that was down-regulated in the brain of HVV rats. On the cellular level, SFP enhanced the expression of NRF2, reduced the concentrations of MDA, GSH and SOD, and promoted the production of NO and cGMP in a dose-dependent manner. In this study, we treated an animal model of HVV with SFP to investigate its effect on NO production and oxidative stress. Our work provided a mechanistic insight into the therapeutic effect of SFP on the treatment of HVV.

## Introduction

As a type of commonly observed symptoms in clinical settings, vertigo is represented by the illusion of body movement induced by the imbalance in the vestibular system [1–3]. In patients suffering from chronic dizziness, more than a quarter of patients show symptoms of vestibular vertigo (VV), which is one of the most frequent symptoms of dizziness around the world [4]. Past researches have shown that an elevated level of oxygen species (OS) is associated with higher risk of vertigo [5–7].

As a type of natural isothiocyanate compound, SFP is best known for its role in protecting against a wide range of pathological disorders [8,9]. SFP can interact with Cysteine located in the Keap1 protein to form a thionoacyl structure while at the same time preventing the degradation of nuclear factor (erythroid-derived 2)-like 2 (Nrf2), so as to promote the nuclear translocation of SFP as well as its binding to anti-oxidant response elements (AREs) [10–12].

As a type of free radicals, nitric oxide (NO) is produced naturally via electrical discharges such as lightning, or synthesized as an essential intermediate in chemical production [13–16]. As a type of factors synthesized endogenously by the endothelium, NO was proposed to exert anti-oxidative effects in the human body in the studies of Robert et al. and Louis et al [9–11], which suggested the negative relationship between NO production and the level of oxidative stress.

The transcription factor Nrf2 encoded by the gene NFE2L2 can control the expression level of many genes involved in a diverse range of protective roles in the cells. For example, the expression level of Nrf2 itself can affect the stability of proteins. In addition, Nrf2 can also be degraded by a Cul1-based ubiquitin ligase dependent on β-TrCP and glycogen synthase kinase (GSK) 3 [17,18]. Furthermore, it was shown that in the presence of stress in the endoplasmic reticulum, Nrf2 can be degraded by Hrd1, an E3 ubiquitin ligase [19]. Also, Nrf2 has been reported as an emerging regulator of cellular resistance to oxidants, which suggested the negative relationship between Nrf2 expression and the level of oxidative stress [20]. Moreover, in PC12 cells, NO has been demonstrated to activate Nrf2 by S-nitrosylation of Keap1 and alternatively by PKC-catalyzed phosphorylation of Nrf2 [21,22].

It has been reported that the activation of NRF2 could lead to the suppressed oxidative stress. Meanwhile, SFP treatment represses oxidative stress by activating NRF2 [23]. Meanwhile, SFP may also increase the production of NO and activate the signaling pathway of cGMP [23]. In respect to the fact that both oxidative stress and NO production are involved in the pathogenesis of HVV, we hypothesized that SFP may play a therapeutic role in the treatment of HVV via modulating oxidative stress and NO production. In this study, we established an animal model of HVV, and administrated the HVV animals with SFP to investigate its effect on NO production and oxidative stress.

## Materials and methods

### Animals, grouping and treatment

In this research, male Sprague-Dawley (SD) rats (average age: 5 weeks ± 3 days, average body weight: 210 ± 10 g) were purchased from our animal center and included in this study. Upon delivery, all rats were placed under 20 ~ 25°C and 50% ± 20% humidity. The light/dark cycle was set to 12 h/12 h. Moreover, all animals had unlimited access to food as well as drinking water. After 7 days of an adaptation period, the animals were randomly divided into 4 groups, i.e., 1. SHAM group (rats undergoing sham operations); 2. SHAM + SFP group (sham operated animals treated with SFP); 3. hypoxic vestibular vertigo group (rats suffering from hypoxic vestibular vertigo); 4. HVV + SFP group (hypoxic vestibular vertigo rats treated with SFP). In the SFP groups, the dose of SFP was given via intraperitoneal administration. To induce hypoxic vestibular vertigo, the rats in the corresponding groups were first placed under anesthesia by using 0.35 ml/100 g of chloral hydrate. Then, the right common carotid artery (CCA) as well as subclavian arteries (SCA) in each rat was exposed and ligated.

### Assay of SOD activity in the aorta

The SOD activity in the aorta was evaluated using a previously reported method [24]. The activity of SOD was represented by U/g of wet tissues., and one unit of SOD activity was represented by the amount of the enzyme causing 50% inhibition of auto-oxidation of pyrogallol.

### Assay of total content of nitrite and nitrate (NOx) in the homogenate of aortic tissues

The total content of nitrate and nitrite in aortic tissues as well as stable metabolite of NO was assayed using a previously reported method [25,26]. In brief, a commercially available experimental kit (R and D System, Minneapolis, MN) was utilized according to the general assay protocol provided by the manufacturer, and the results of nitrate contents in the homogenate of aortic tissues were converted to the contents of nitrite.

### Aortic homogenate preparation

A 5% w/v homogenate of aortic tissues was prepared by utilizing a small homogenizer (Omni international, Tulsa, OK). After the homogenates of aortic tissues were obtained, they were centrifuged for 10 min at 1000 g and 4℃, and the supernatant was collected for following assays.

### Assay of MDA level in aortic tissues

The level of lipid peroxidation in the supernatant of tissue and cell samples was assayed by utilizing an assay kit of thiobarbituric acid reactive reagents according to the general assay protocol provided by the manufacturer. In brief, 200 mL of homogenate of aortic tissues were mixed with 2 mL of a 20% solution of acetic acid (pH = 3.5), 200 mL of 8% SDS, as well as 2 mL of an aqueous solution containing 0.8% of TBA. In the next step, the mixture was incubated for 60 min in an incubator at 95 C, cooled down, diluted with distilled water, and then centrifuged for 10 min at 1200 g before the OD value of each sample was measured at 532 nm by utilizing a plate reader (Bio-Rad, Hercules, CA).

### Assay of GSH level in aortic tissues

The levels of thiols soluble in acids, i.e., GSH, in the supernatant of tissue and cell samples were assayed by utilizing a colorimetry assay kit according to the general assay protocol provided by the manufacturer.

### RNA isolation and real-time PCR

To assay the expression of NRF2 mRNA in collected tissue and cell samples, total RNA was first isolated from each sample by utilizing a Trizol reagent kit (Invitrogen, Carlsbad, CA) according to the general assay protocol provided by the manufacturer. In the next step, the isolated RNA samples were converted into cDNA templates by using reverse transcription carried out by utilizing a qScript cDNA synthesis kit (Quanta Biosciences, Gaithersburg, MD) according to the general assay protocol provided by the manufacturer. Then, quantitative real time PCR was carried out on a Light Cycler 480 real time PCR apparatus (Roche, Manheim, Germany) by utilizing a SYBR Green I Master kit (Roche, Manheim, Germany) according to the general assay protocol provided by the manufacturer. Finally, the relative expression of NRF2 in each sample was calculated using the calculated Ct values based on the 2^−ΔΔCt^ method [27].

### Cell culture and transfection

We utilized a neuroblastoma cell line, SH-SY5H, to perform the cell studies. SH-SY5H cells have been used and reported in the study of CNS diseases. In this study, SH-SY5H cells were acquired from Shanghai Cell Bank, the Chinese Academy of Sciences (Shanghai, China). The cells were maintained by using a standard DMEM medium (Gibco, Thermo Fisher Scientific, Waltham, MA) containing 10% of fetal bovine serum (Hyclone, Thermo Fisher Scientific, Waltham, MA), 20 mmol/L glucose, 100 μg/mL streptomycin, 100 U/mL penicillin, as well as appropriate antibiotics. The cell culture conditions were 5% CO2, saturated humidity, 95% air, and 37°C. After being cultured for 12 h, the cells were randomly divided into 3 groups, i.e., 1. Untreated group (cells grown in regular DMEM); 2. 2.5 μmol/L SFP group (cells grown in regular DMEM containing 2.5 μmol/L of SFP); and 3. 5 μmol/L SFP group (cells grown in regular DMEM containing 5 μmol/L of SFP. The centrations of SFP (2.5 μmol/L and 5 μmol/L) were determined by examining the effect of different concentrations of SFP in our preliminary test (Supplementary Figure 1). After 72 h of cell culture, the cells were collected for subsequent analysis.

### Western blot analysis

The expression of NRF2 proteins in collected rat brain tissues and cultured cells was assayed using a Western blot analysis. In brief, the tissue and cell samples were lysed and homogenized, and protein supernatant was collected after centrifugation. In the next step, 20 μg of isolated protein in each sample were resolved by using 12% SDS-PAGE, and then transferred onto a nitrocellulose (NC) membrane (Thermo Fisher Scientific, Waltham, MA), which was then blocked by using PBS containing 0.1% of TBST and 7% of nonfat milk. After being incubated in a 4 C fridge overnight with primary anti-NRF2 antibodies (Abcam, Cambridge, MA) according to the protocol provided by the antibody manufacturer, the membrane was further incubated at room temperature for 2 hr by using HRP-conjugated secondary antibody before it was visualized and analyzed by utilizing an imaging system equipped with ImageJ software.

### Immunohistochemistry

The expression of NRF2 proteins in collected rat brain tissues was assayed using a routine immunohistochemistry assay. In brief, the collected rat brain tissues were prepared into 5 μm thick sections, fixed in paraformaldehyde, embedded in paraffin, de-paraffinized by using xylene, re-hydrated by using gradient ethanol, subjected to antigen retrieval by boiling in a pH 6.0 citrate buffer for 1 hr at 96°C, and then treated by utilizing a Polymer novolink kit (Leica, Nanterre, France) according to the general protocol provided by the manufacturer. After being incubated at 4°C overnight by using anti-NRF2 primary antibody (1:8000 dilution in PBS, Abcam, Cambridge, MA) as well as corresponding secondary antibodies, the slides were counter stained using a DAB substrate and the signal of positive NRF2 expression was detected by using a microscope (IM 1000, Leica, Nanterre, France).

### ELISA assay

The levels of NO and cGMP in collected rat brain tissues as well as cultured cells were determined by utilizing commercial available ELISA assay kits (eBioscience, San Diego) according to the general assay protocol provided by the manufacturer.

### Statistical analysis

All data was analyzed by utilizing GraphPad Prism 7.0 software. All comparisons among different groups were carried out by utilizing analysis of variance (ANOVA) and Student’s *t*-test. A *p* value of < 0.05 was deemed statistically significant.

## Results

### The increased escape latency of HVV rats was reduced by SFP treatment

In this study, we hypothesized that SFP may play a therapeutic role in the treatment of HVV via modulating oxidative stress and NO production. In this study, we established an animal model of HVV, and administrated the HVV animals with SFP to investigate its effect on NO production and oxidative stress.

**Figure 1.**
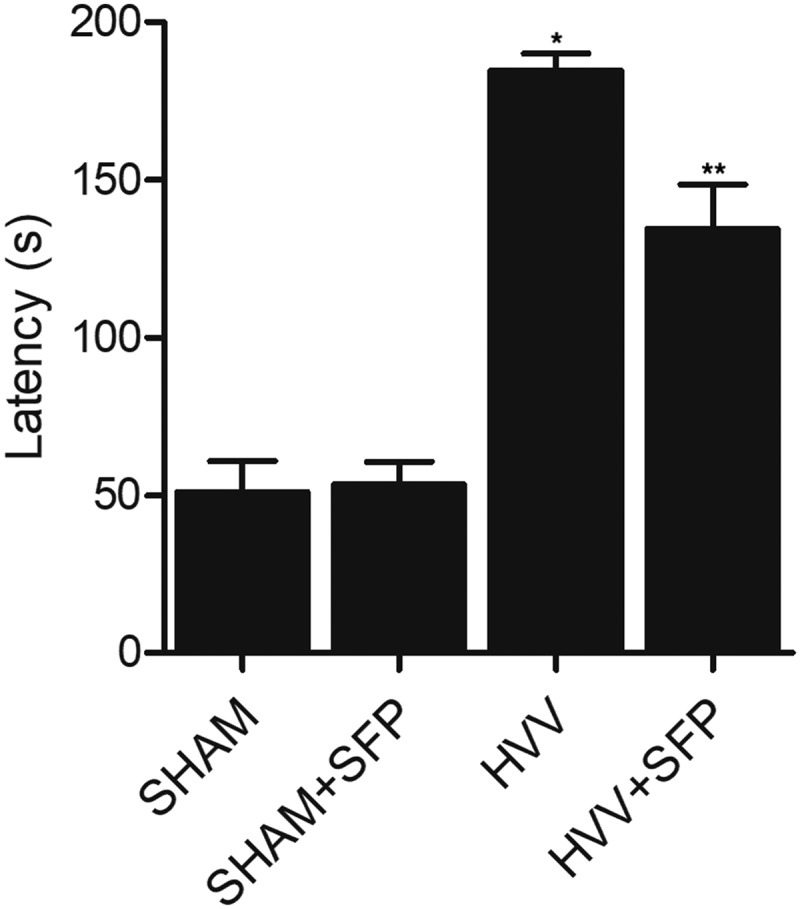
The increased escape latency of HVV rats was reduced by SFP treatment (N = 3; * P value < 0.05, vs. SHAM group; ** P value < 0.05, vs. HVV group).

The escape latency of rats treated under different conditions was evaluated. As shown in Figure 1, the escape latency of HVV rats was significantly elevated when compared with that in the SHAM control rats. Treatment with SFP notably decreased the escape latency of HVV rats, but showed no effect on the SHAM control rats (Figure 1).

### SFP treatment maintained the levels of MDA/SOD and GSH in the aorta tissues of HVV rats

**Figure 2.**
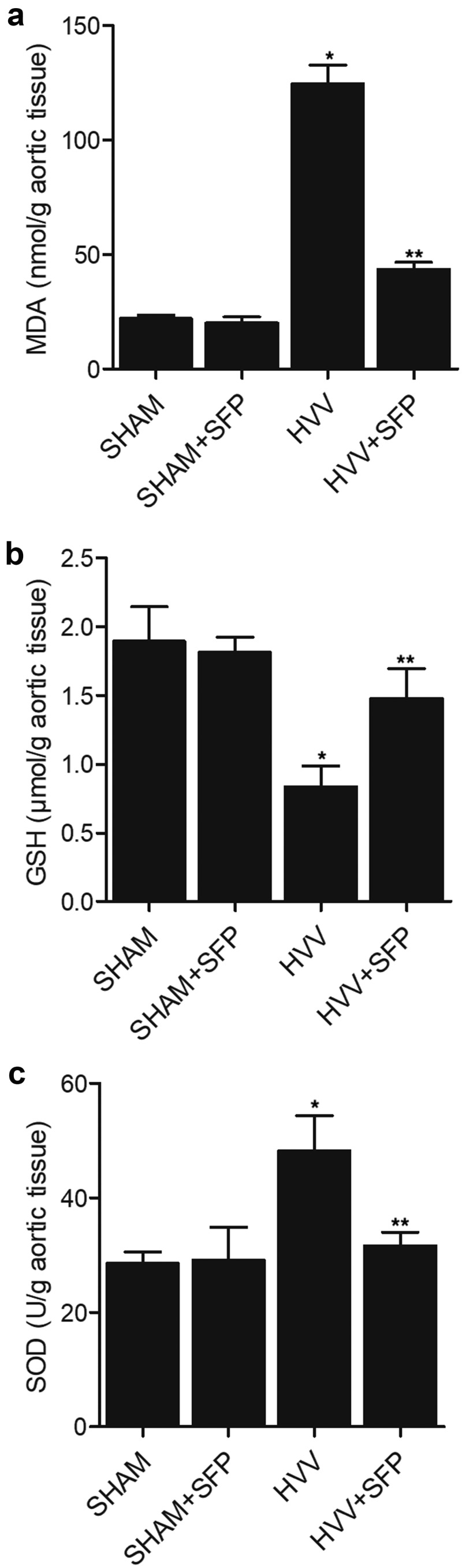
SFP treatment could maintain the concentrations of MDA, GSH and SOD in the aorta of rats (N = 3; * P value < 0.05, vs. SHAM group; ** P value < 0.05, vs. HVV group).

In order to further explore the effects of SFP treatment on oxidative stress, we harvested the aorta tissues of rats and analyzed the contents of MDA, GSH and SOD in rats treated under different conditions. The concentrations of MDA and SOD in the aorta tissues were remarkably increased in HVV rats. Treatment with SFP efficiently recovered the levels of MDA (Figure 2(a)) and SOD (Figure 2(c)) in the aorta tissues of HVV rats, but showed no obvious effects in the SHAM control rats. On the contrary, the concentration of GSH in the aorta tissue of HVV rats was obviously reduced. SFP treatment restored the level of GSH in the aorta tissues of HVV rats (Figure 2(b)).

(a) Aberrantly increased MDA concentration in the aorta of HVV rats was restored by SFP treatment.(b) Aberrantly reduced GSH concentration in the aorta of HVV rats was restored by SFP treatment.(c) Aberrantly increased SOD concentration in the aorta of HVV rats was restored by SFP treatment.

### SFP treatment restored the level of NRF2 expression in the brain of HVV rats

**Figure 3.**
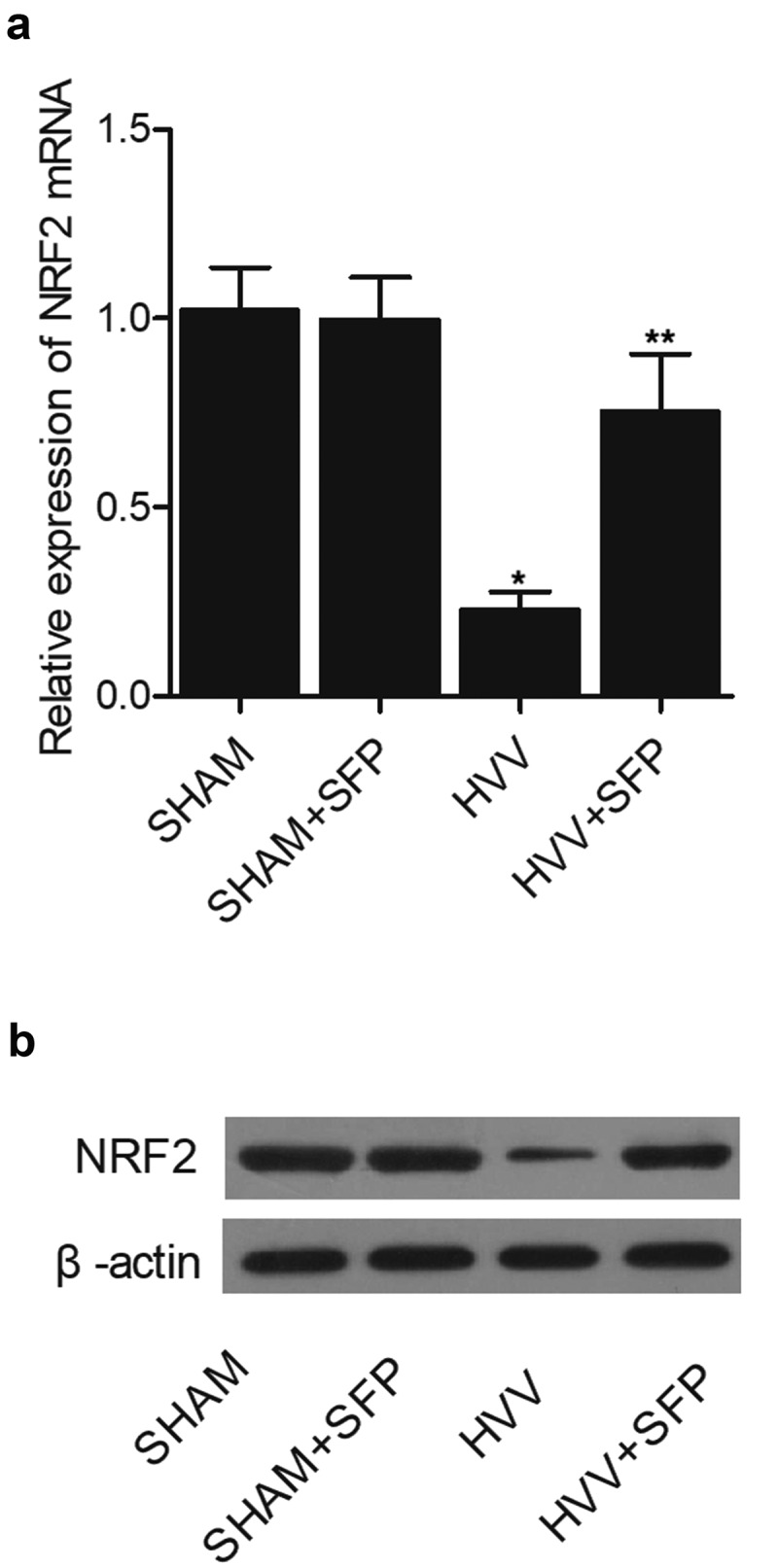
Suppressed expression of NRF2 mRNA and protein in the brain of HVV rats was restored by SFP treatment (N = 3; * P value < 0.05, vs. SHAM group; ** P value < 0.05, vs. HVV group).

**Figure 4.**
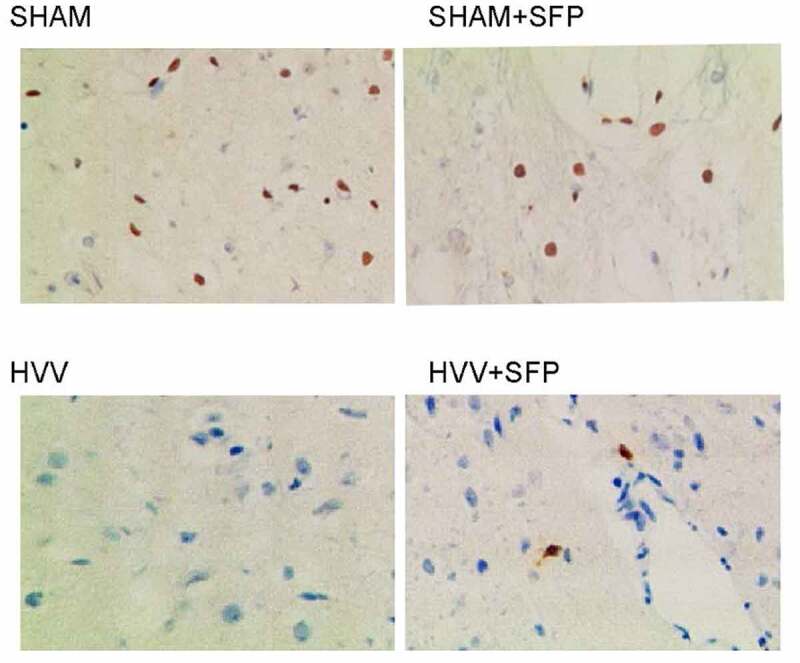
Immunohistochemistry analysis indicated that the expression of NRF2 protein in the brain of HVV rats was restored by SFP treatment.

NRF2 is an emerging regulator of cellular resistance to oxidants in the response to oxidative stress. In this study, we examined the expression of NRF2 mRNA and protein in the brain tissues of rats treated under different conditions: 1. SHAM control, 2. SHAM + SFP, 3. HVV, 4. HVV + SFP. Quantitative real-time PCR was performed to assess the expression of NRF2 mRNA, which was notably inhibited in the brain tissues of HVV rats but recovered by the treatment with SFP (Figure 3(a)). Similarly, the expression of NRF2 protein was also repressed in the brain of HVV rats and restored by SFP treatment, as revealed by Western blot (Figure 3(b)) and immunohistochemistry (Figure 4).

(a) SFP treatment recovered the NRF2 mRNA expression in the brain of HVV rats.(b) SFP treatment recovered the NRF2 protein expression in the brain of HVV rats.

### SFP treatment recovered the level of NO and cGMP production in the brain of HVV rats

**Figure 5.**
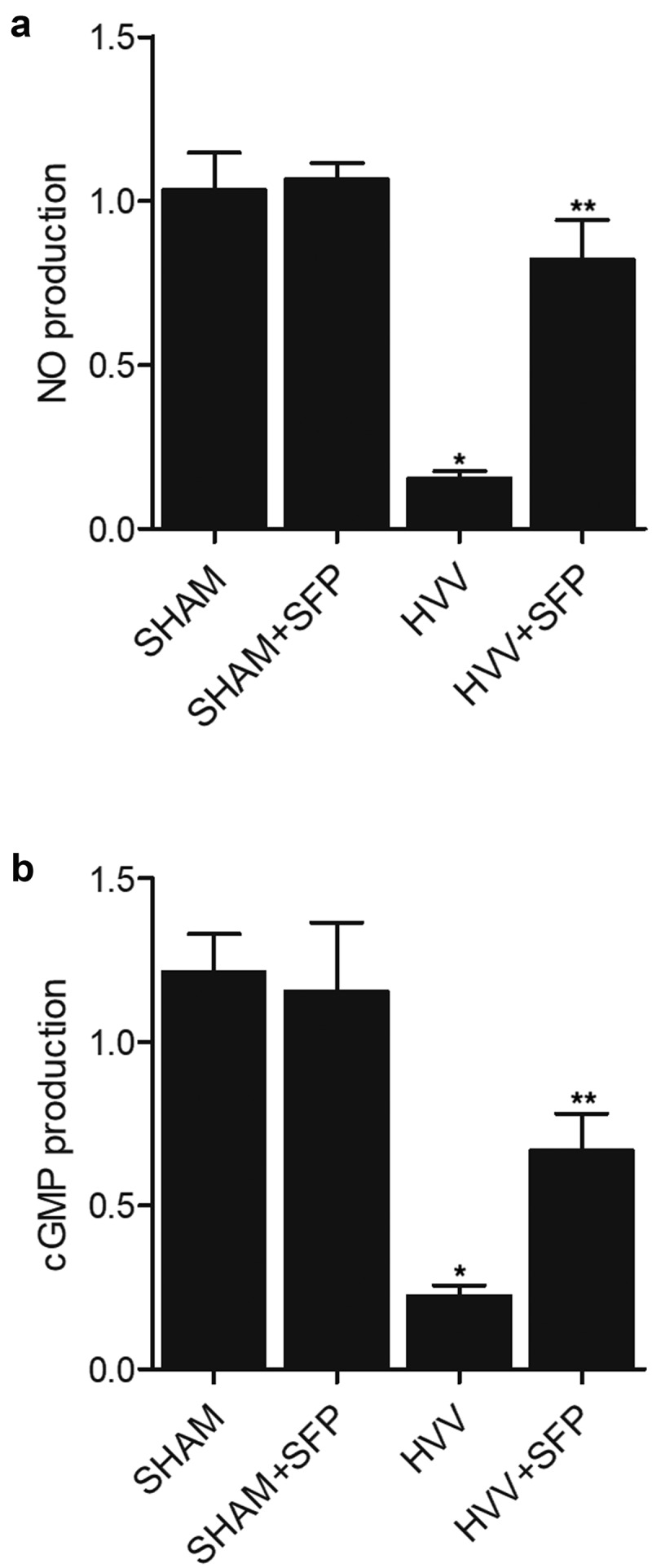
Declined production of NO and cGMP in the brain of HVV rats was restored by SFP treatment (N = 3; * P value < 0.05, vs. SHAM group; ** P value < 0.05, vs. HVV group).

NO and cGMP production was key biomarkers that are negatively associated with the response to oxidative stress. We found that NO production in the brain tissues of HVV rats was dramatically suppressed, and the treatment with SFP could efficiently up-regulate NO production in the brain of HVV rats (Figure 5(a)). Moreover, the production of cGMP was also repressed in the brain tissues of HVV rats, and the SFP treatment effectively restored the production of cGMP in the brain of HVV rats (Figure 5(b)).

(a) NO production decreased in the brain of HVV rats was restored by SFP treatment.(b) cGMP production decreased in the brain of HVV rats was recovered by SFP treatment.

### SFP treatment enhanced the expression of NRF2 mRNA and protein in SH-SY5H cells in a dose-dependent manner

**Figure 6.**
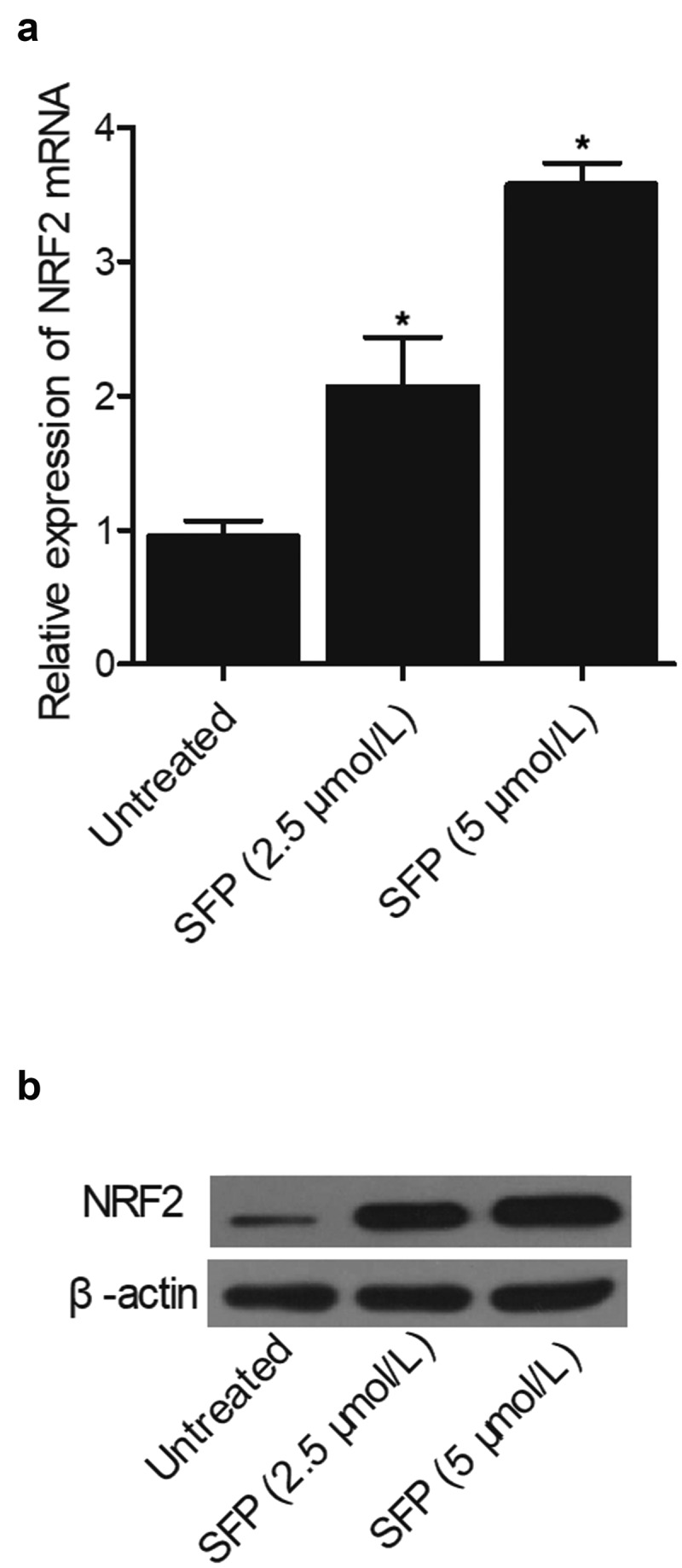
SFP activated the expression of NRF2 in SH-SY5H cells in a dose-dependent manner (N = 3; * P value < 0.05, vs. untreated group).

To further confirm the therapeutic effect of SFP on the cellular level, SH-SY5H cells were treated with SFP at different concentrations: 2.5 μmol/L and 5 μmol/L. Next, the expression of NRF2 mRNA and protein was measured with qPCR and Western blot. We found that the expression of NRF2 mRNA (Figure 6(a)) and protein (Figure 6(b)) was remarkably up-regulated by SFP at both 2.5 μmol/L or 5 μmol/L, indicating that SFP treatment could elevate the expression of NRF2 in a dose-dependent manner.

(a) NRF2 mRNA expression in SH-SY5H cells was enhanced by an increasing concentration of SFP.(b) NRF2 protein expression in SH-SY5H cells was enhanced by an increasing concentration of SFP.

### SFP treatment decreased the concentrations of MDA and SOD while increasing the concentration of GSH and production of NO and cGMP in a dose-dependent manner

**Figure 7.**
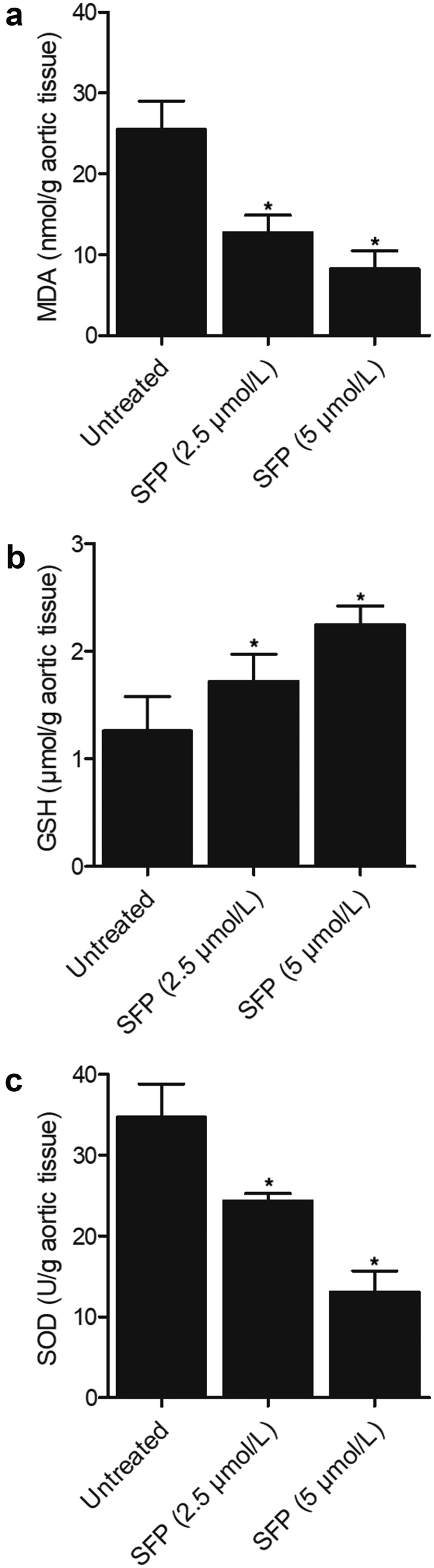
Concentrations of MDA, GSH and SOD in the SH-SY5H cells were reduced by SFP treatment in a dose-dependent manner (N = 3; * P value < 0.05, vs. untreated group).

**Figure 8.**
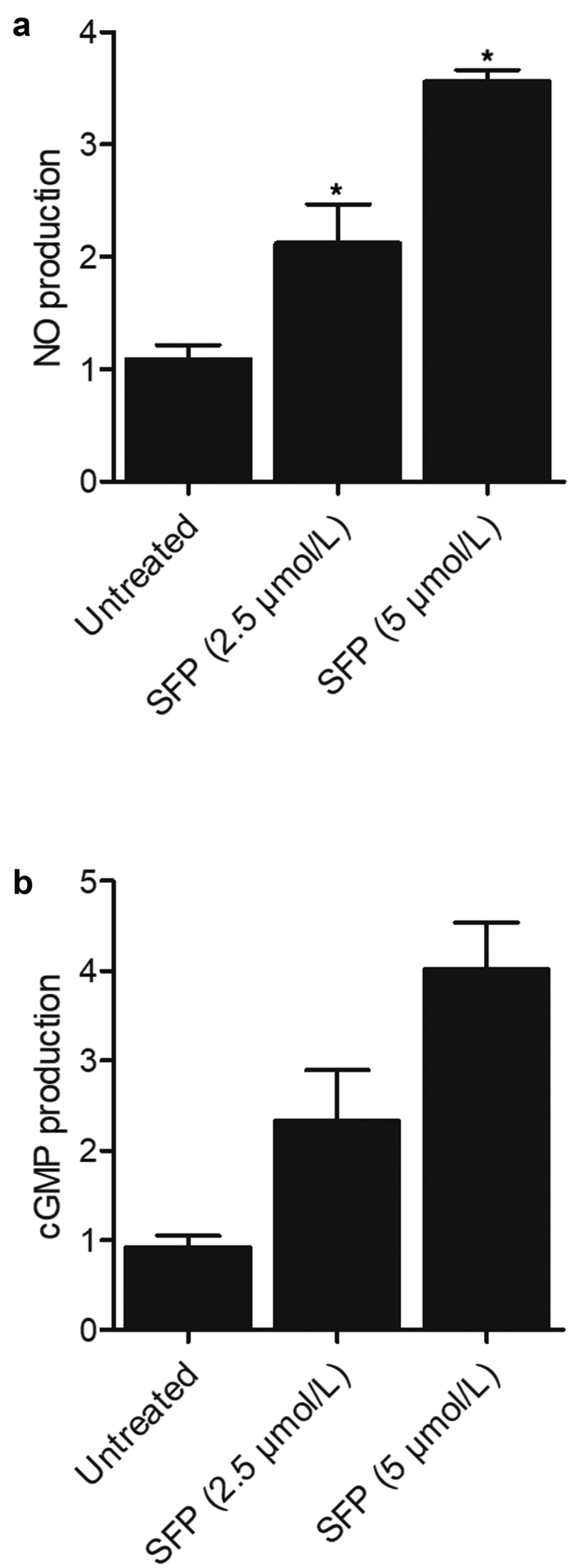
Production of NO and cGMP in SH-SY5H cells was enhanced by SFP treatment in a dose-dependent manner (N = 3; * P value < 0.05, vs. untreated group).

The concentrations of MDA (Figure 7(a)) and SOD (Figure 7(c)) in SH-SY5H cells were significantly decreased by SFP treatment at a concentration of 2.5 μmol/L, and the effect of SFP was even stronger at 5 μmol/L in SH-SY5H cells. On the other hand, the concentration of GSH (Figure 7(b)) in SH-SY5H cells, as well as the production of NO (Figure 8(a)) and cGMP (Figure 8(b)) in SH-SY5H cells, was efficiently activated by SFP treatment in a dose-dependent manner.

(a) The concentration of MDA was reduced in SH-SY5H cells by SFP in a dose-dependent manner.(b) The concentration of GSH was promoted in SH-SY5H cells by SFP in a dose-dependent manner.(c) The concentration of SOD was reduced in SH-SY5H cells by SFP in a dose-dependent manner.

(a) NO production in SH-SY5H cells was enhanced by SFP treatment in a dose-dependent manner.(b) cGMP production in SH-SY5H cells was enhanced by SFP treatment in a dose-dependent manner.

## Discussion

As a type of isothiocyanates generated by the hydrolysis of glucosinolate glucoraphanin, SFP was shown to increase the expression level of proteins with cytoprotective roles via the signaling of Nrf2 transcription factor [28]. SFP can also play anti-tumor, anti-oxidant, as well as anti-inflammatory roles [28]. In addition, SFP exerts a certain neuroprotective effect both in vivo and in vitro [29]. Past research demonstrated that SFP treatment apparently reduced the levels of NO in the lung of mouse suffering from ALI induced by LPS. Brandenburg and his colleagues [30]* showed that SFP reduced the inflammation triggered by LPS via reducing the levels of NO [31]. In this study, we established a HVV rat model and evaluated the escape latency of rats treated under different conditions. The escape latency of HVV rats was significantly elevated when compared with the control rats, and SFP treatment restored the escape latency in HVV rats. In addition, we measured the concentrations of MDA, GSH and SOD in the brain of HVV rats. We found that MDA and SOD concentrations were notably increased in the brain of HVV rats, but SFP treatment efficiently recovered the concentrations of MDA and SOD in HVV rats. However, the changes of GSH concentration in rats treated under different conditions showed an opposite trend.

As a type of free radicals ubiquitously present among a wide range of cells, NO can be synthesized by endothelial nitric oxide synthase (eNOS, also termed NOS-3), neuronal nitric oxide synthase (nNOS, also termed NOS-1), as well as inducible nitric oxide synthase (iNOS, also termed NOS-2) [32]. NO can stimulate its receptor sGC present in the cells, which in turn converts GTP into cGMP to activate phosphodiesterases (PDEs), cyclic nucleotide-gated ion channels, as well as cGMP-dependent protein kinases. In fact, as a second messenger, cGMP is generated under the action of guanylyl cyclases (GC), whose membrane bound form pGC can be activated by brain natriuretic peptides (BNP), atrial natriuretic peptides (ANP), as well as C-type natriuretic peptides (CNP) [33]. In this study, we performed ELISA to evaluate the production of NO and cGMP in the brain of HVV rats treated under different conditions. NO and cGMP production was remarkably repressed in the brain of HVV rats, and SFP treatment recovered the levels of NO and cGMP production.

Past research showed that the expression level of Nrf2 in the cardiomyocytes of neonatal rats can be increased by SFP when the cardiomyocytes are damaged by H2O2 [34]. In addition, SFP can partially prevent cardiomyopathy through the signaling of Akt/GSK-3β/Fyn and activation of Nrf2 [35]. In this study, we analyzed the expression of NRF2 mRNA and protein in the brain of HVV rats treated under different conditions. NRF2 expression was significantly inhibited in the brain of HVV rats, and SFP treatment could up-regulate the expression of NRF2 mRNA and protein in the brain of HVV rats. In addition, Nrf2 is considered as a critical regulator in the functions of antioxidation and detoxification proteins. Initially discovered as a protein able to bind to the globin gene, Nrf2 is expressed constitutively by nearly all types of cells, especially in the kidney, lungs and intestine [36,37]. Nrf2 plays its protective role against oxidative damages by interacting with antioxidant response element (ARE) in its target genes [38]. In the lungs, the target genes of Nrf2 include GPx, SODs, catalases, as well as enzymes in the thiol-redox system including Prx, GCS, GST isozymes, UGT isozymes, and stress proteins [39].

Nrf2 not only participates in the metabolism process of calcium, but also has a close relationship with the level of oxidative stress in the body. For example, by studying the potential role of angiitis as well as diacron reactive oxygen metabolites (d-ROM) in the pathogenesis of benign paroxysmal positional vertigo (BPPV), Goto and his colleagues showed that a higher level of d-ROM as well as vascular cell adhesion molecule-1 (VCAM-1) was present among BPPV patients suffering from chronic vertigo attacks [7]. As a result, the authors believed that angiitis as well as ROM participate in BPPV pathogenesis [40].

In this study, we treated SH-SY5H cells with different concentrations of SFP and examined the expression of NRF2, the concentrations of MDA, GSH and SOD, and the production of NO and cGMP. Our results demonstrated that SFP treatment played a regulatory role in the expression of NRF2, the concentrations of MDA, GSH and SOD, and the production of NO and cGMP in the SH-SY5H cells in a dose-dependent manner.

## Conclusion

In summary, our findings suggest that SFP can improve NO production and reduce the expression of NRF2 in HVV animal models, possibly by inhibiting the production of cGMP and NRF2 while reducing the activity of SOD, GSH, and MDA.

## Supplementary Material

Supplemental Material

## Data Availability

The data that support the findings of this study are available from the corresponding author upon reasonable request.
