# Supplementary figures and images for: Sulforaphane alleviates hypoxic vestibular vertigo (HVV) by increasing NO production via upregulating the expression of NRF2
Source: Bioengineered. 2022 Apr 20;13(4):10351–61. doi: 10.1080/21655979.2022.2030592 (PMC9161921; doi:10.1080/21655979.2022.2030592)

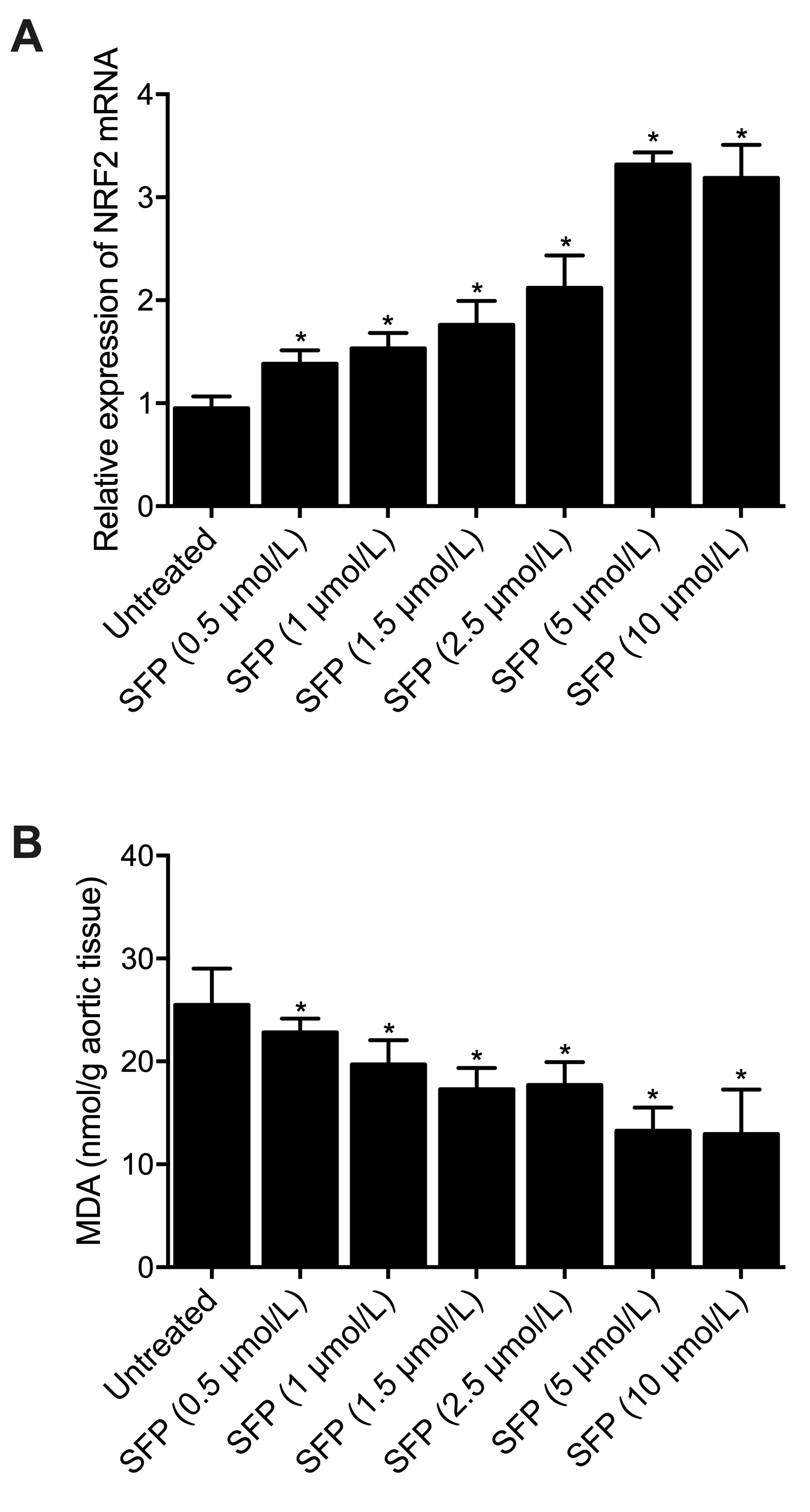

Supplement: Supplemental Material [file KBIE_A_2030592_SM1253.tiff]
